# The Use of Pyrolytic Char Derived from Waste Tires in the Removal of Malachite Green from Dyeing Wastewater

**DOI:** 10.3390/nano12234325

**Published:** 2022-12-05

**Authors:** Dongliang Ji, Didi Gai, Yikun Xu, Zhaoqin Huang, Peitao Zhao

**Affiliations:** 1 College of Environment and Ecology, Jiangsu Open University, Nanjing 210036, China; 2 School of Low-Carbon Energy and Power Engineering, China University of Mining and Technology, Xuzhou 221116, China

**Keywords:** waste tire, pyrolytic char, adsorption, malachite green, metal oxide catalyst

## Abstract

The organic dye malachite green (MG) poses a potential risk of cancer and fertility loss in humans and aquatic organisms. This study focused on a modified pyrolytic char (PC) derived from waste tires to efficiently remove MG from wastewater. Modified PC has rich -OH functional groups, higher BET (Brunauer-Emmett-Teller) surfaces of 74.4, 64.95, and 67.31 m^2^/g, and larger pore volumes of 0.52, 0.47, and 0.62 cm^3^/g for NaOH, Na_2_CO_3_, and CaO modification, respectively. The pseudo-second-order model fit the adsorption well, and the maximum equilibrium adsorption capacity was 937.8 mg/g for PC after CaO activation (CaO-PC). NaOH-modified PC (NaOH-PC) showed the best fit with the Langmuir model (R^2^ = 0.918). It is suggested that alkali-modified waste tire pyrolytic char could be a potential adsorbent for removing MG from dye-containing wastewater.

## 1. Introduction

Water pollution [1] has become an increasingly serious problem to be solved due to the increase in population and the development of the world economy [2]. Numerous pollutants and poisons are discarded into water because of human negligence and ignorance, causing the destruction of aquatic ecosystems and the reduction of clean water resources [3,4,5]. Among these, wastewater from printing and dyeing is a substantial source of water pollution. Its presence not only pollutes water resources, but also causes non-degradable toxicity, which can cause cancer, malformation, and even affect fertility [5]. Malachite green (MG) is a basic dye used in cotton, silk, leather, and paper dyeing [5,6,7,8,9]. It is also used in aquaculture for sterilization [10], which is illegal in some countries owing to its harmful effects on water quality. Adsorption is the most effective method for wastewater treatment [11]. Activated carbon is the most commonly used material for adsorbing anions and cations in water because of its high adsorption capacity, fast adsorption kinetics, and relative ease of regeneration. However, the commercial production of activated carbon for adsorption is limited owing to its expensive raw materials (such as coal) and non-renewable use [12]. For adsorption to be applied to wastewater on a larger scale, the development of cheap and accessible adsorbent raw materials is essential.

A large number of waste tires are produced with the continuous development of the transportation industry, with 13.5 million tons of waste tires produced every year [13]. The waste tires from cars (without metal and fabric cords) are composed of rubber (60–65%), carbon black (approximately 30%), plasticizers (7.5%), and vulcanizing agents (3–5%) [14]. Discarded waste tires can become breeding grounds for mosquitoes and bacteria. Moreover, the unique physiochemical properties of waste tires do not allow for easy degradation [15]. Their large size makes them unsuitable for landfill treatment, and their combustion can release harmful and polluting gases [16,17]. Therefore, the recycling and utilization of waste tires have attracted the attention of people worldwide over the possible production of adsorbents and value-added products using thermochemical processes to realize the step utilization of waste tires [18].

Pyrolysis has been considered a good way to obtain bio-oil, syngas, and char from waste tires as it has less negative environmental impact than combustion and gasification [13,19]. The main component of pyrolytic char (PC) is black carbon from tires, which accounts for approximately 90% of the solid product [14]. It contains numerous carbon elements, and its properties can be improved by physical or chemical methods to obtain a porous material that can be used as a catalyst support or adsorbent [20]. The adsorbents derived from waste tire PC have been investigated for the adsorption of hazardous substances from liquid [21,22,23,24,25,26] and gas phases [20,27]. The results showed that the adsorption capacity of waste tire PC after activation was similar to that of commercial activated carbon. Various activators have been explored to achieve the ideal textural and surface chemical properties [18,28,29]. The main activation methods consist of physical and chemical activation [9]. Jusoh et al. [30] prepared waste tire char at three calcination temperatures (500 °C, 700 °C, 900 °C) and then activated the solution with NaOH to remove paracetamol; the highest removal percentage (99.37%) was obtained for 900 °C calcinated carbon in the conditions of an initial concentration of 10 mg/L, a pH of 3, and 120 min of contact time. Waste tire char was employed as the precursor and activated by steam to obtain well-developed mesoporous carbons with a burn-off of approximately 43% (TC43). TC43 has been applied in methylene blue (MB) removal and showed the highest adsorption amount of 250 mg/g for a 50 mL MB solution with an initial concentration of 1200 mg/L at a temperature of 50 °C [31]. Özbaş et al. [32] activated end-of-life tires using KOH solutions with ratios of 1:1 and 1:2 to obtain activated carbon with a BET (Brunauer-Emmett-Teller) surface area of 2.945 m^2^/g. Single-activation methods, such as pure chemical or physical activation, have higher requirements for reaction conditions or require a secondary reaction. Several studies have reported that some basic additives, mainly metal oxides [33], can improve pyrolysis and be helpful for pore formation [34,35,36,37].

This study intended to reveal the feasibility of utilizing pyrolytic char to remove MG from dye-containing wastewater and to evaluate the effect of this chemical modification on the adsorption performance. For these purposes, waste tires were slowly pyrolyzed with and without the addition of NaOH, CaO, and Na_2_CO_3_. N_2_ adsorption-desorption, X-ray photoelectron spectroscopy (XPS), and Fourier transform infrared spectroscopy (FT-IR) were used to characterize the structural properties and surface chemistry of the PCs. The adsorption capacity was evaluated at various pH values, initial MG concentrations, and equilibrium adsorption times. The experimental adsorption data were analyzed using adsorption kinetics and isotherms to explore the adsorption pathways and mechanisms. This study provides guidance for the highly efficient recycling of waste tires and the development of a circular economy.

## 2. Materials and Methods

### 2.1. Materials

The tires used in this study were purchased from a local tire waste market. They were crushed and cut into pieces with diameters of approximately 5 mm. The basic elemental composition of the raw materials is listed in Table 1.

### 2.2. Preparation of Pyrolytic Char

**Figure 1:**
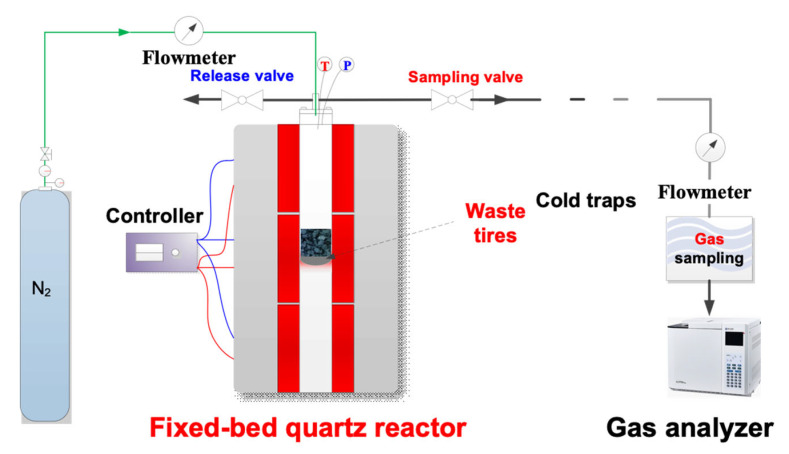
Schematic diagram of tire pyrolysis.

Waste tires (100 g) were placed in the reactor, which was then covered and placed in a pyrolysis furnace, as shown in Figure 1. The starting temperature of the inner furnace was maintained at 20 °C during the pyrolysis process. Temperature programming for the pyrolysis was set to increase from 20 °C to 450 °C for 35 min, then remain steady at 450 °C for 25 min to release oil and gases. Before the temperature of the reactor reached 120 °C, the release valve was opened and the sampling valve on the right side was closed to remove the moisture. When the reactor temperature reached 120 °C, the release valve was closed and the sampling valve opened to collect the oil and gases. Bio-oil was produced as the condensate, which was discussed in separate research. The non-condensing gas at the outlet was first measured using a wet flowmeter and gathered in a gas sampling bag. After the reaction, the reactor was removed from the furnace and cooled to room temperature using a fan. Finally, the solid product was removed from the reactor and denoted RWT-PC.

For pyrolysis with NaOH, CaO, and Na_2_CO_3_, 100 g of tires was mixed with 4 g NaOH/CaO/Na_2_CO_3._ The mixture was doped evenly and sent to the reactor. The subsequent pyrolysis steps were consistent with the steps of the reaction without the addition of oxides.

### 2.3. Adsorbent Characterization

FT-IR was employed to explore the spectral patterns of the PC and modified PCs derived from waste tires using a pressing potassium bromide troche (Thermo Fisher Scientific, Waltham, MA, USA). The scanning wavelength of the analysis was 4000–400 cm^−1^.

The surface area and pore size were measured using an analyzer (Beishide Instrument, Beijing, China). The specific structural parameters of the analyzer were obtained from the N_2_ adsorption-desorption curves. The specific surface area and pore size distribution were determined using the BET method.

XPS measurements were conducted using a Thermo Scientific ESCALAB 250 XI XPS microprobe. The analyzer had a monochromatic aluminium anode target equipped with a spot size of 900 μm.

### 2.4. Adsorption Procedure

The adsorption capacities of RWT-PC, NaOH-PC, CaO-PC, and PC after Na_2_CO_3_ activation (Na_2_CO_3_-PC) for MG were measured via adsorption experiments. Forty milligrams of X-PC (X = RWT, NaOH, CaO, Na_2_CO_3_) were added to 40 mL of MG solution with an initial concentration of 100–1000 mg/L. The mixture was then oscillated at 130 rpm for 48 h at 301 K for adsorption. After adsorption, the MG solution was filtered and its concentration measured at 618 nm using a UV-1800PC spectrophotometer (Mapada, Shanghai, China). The equilibrium adsorption capacities (*q_e_*) of RWT-PC, NaOH-PC, CaO-PC, and Na_2_CO_3_-PC for MG dyes were calculated using Equation (1).

(1) $$q_{e}=\frac{\left(C_{O}-C_{e}\right)\times V}{W}$$

## 3. Results and Discussions

### 3.1. Physicochemical Characteristics

#### 3.1.1. Functional Groups Analysis

**Figure 2:**
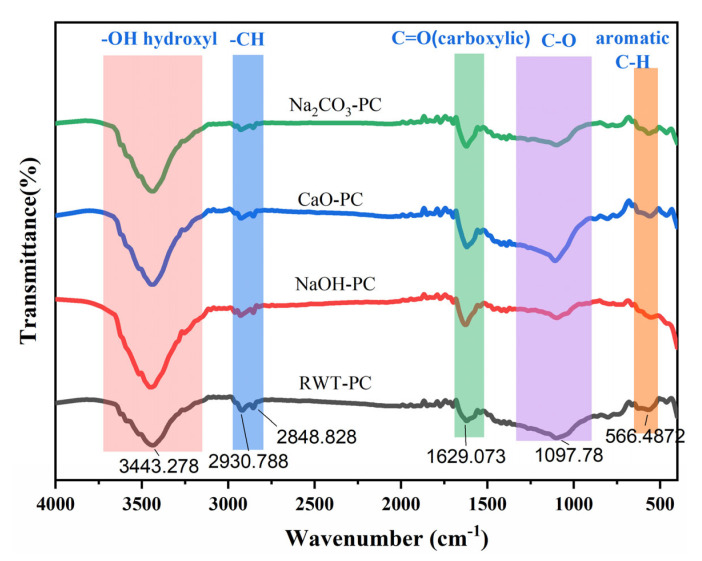
Surface chemical properties of pyrolytic char (PC) derived from waste tires.

The FT-IR spectra showed that all adsorbents from waste tires before and after treatment had abundant chemical functional groups (Figure 2). In the spectrum of different PC adsorbents, the wide band at 3135–3663 cm^−1^ was classified as the -OH stretching vibration of hydroxyl functional groups from alcohols and phenols [38]. The surface of the solid with the catalyst additive had a larger area of integration than that of RWT-PC, which could promote char surface charge generation [39] and further increase the adsorption capacity. The dominant characteristic bands at 2800–2976 cm^−1^ and 495–647 cm^−1^ were assigned to the -CH stretching of the aliphatic and aromatic C-H bending vibrations, respectively [40,41]. The absorption peaks in the range of 1510–1750 cm^−1^ demonstrated the presence of C=O on the adsorbent surface [42,43]. The intensity of the peaks at 1629.073 cm^−1^ followed the order: CaO-PC > NaOH-PC > Na_2_CO_3_-PC > RWT-PC. It was demonstrated that C=O was attributed to hydrophilic functional groups; therefore, the results indicated that the modified PCs exhibited increased surface hydrophilicity, which was beneficial to the adsorption of water-soluble pollutes [44]. The band in the region of 890–1320 cm^−1^ was ascribed to C-O stretching in hydroxyl or ester groups [9,45], implying the existence of a phenolic hydroxyl group [46] and indicating that all the adsorbents had complex surface chemistries.

**Figure 3:**
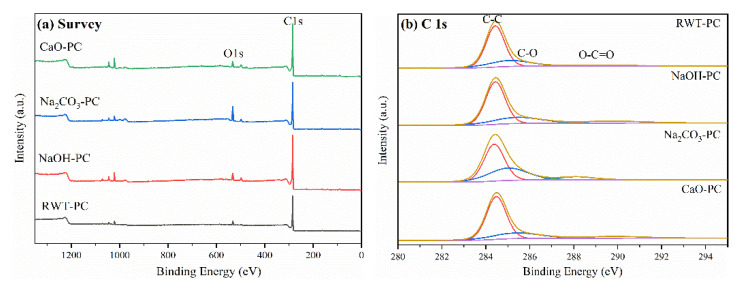
(**a**) Complete X-ray photoelectron spectroscopy (XPS) scan of pyrolytic chars and (**b**) C1s spectrum.

The surface chemical compositions of the adsorbents were further ascertained by XPS, and the spectra are shown in Figure 3. The high-resolution C1s spectrum was deconvoluted into three main peaks at 284.4, 285, and 289 eV. The binding energy of 284.4 eV could be attributed to C-C (sp^2^) in graphene [47]. The peak at approximately 285 eV was ascribed to the C-O bond, while the peak at 288–289 eV was assigned to the C=O bond [48]. The content of oxygen-containing groups increased after metal oxide modification, and NaOH-PC showed the highest C=O bond increase, from 4.41% (RWT-PC) to 9.87% NaOH-PC (Table 2). Hence, the metal oxides in the in situ catalytic pyrolysis of waste tires are beneficial for the formation of oxygen-containing groups, which could promote dye adsorption.

#### 3.1.2. N_2_ Adsorption-Desorption Test

**Figure 4:**
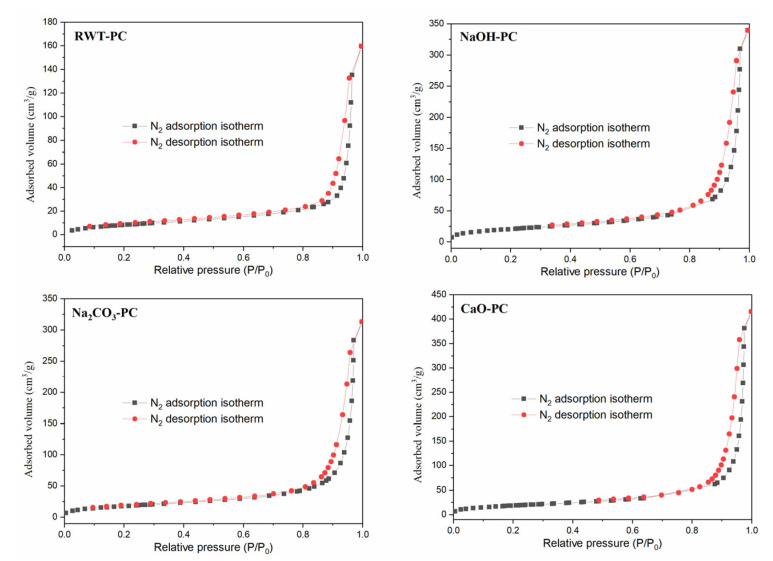
N_2_ adsorption-desorption isotherms of pyrolytic char (PC) derived from waste tires.

**Figure 5:**
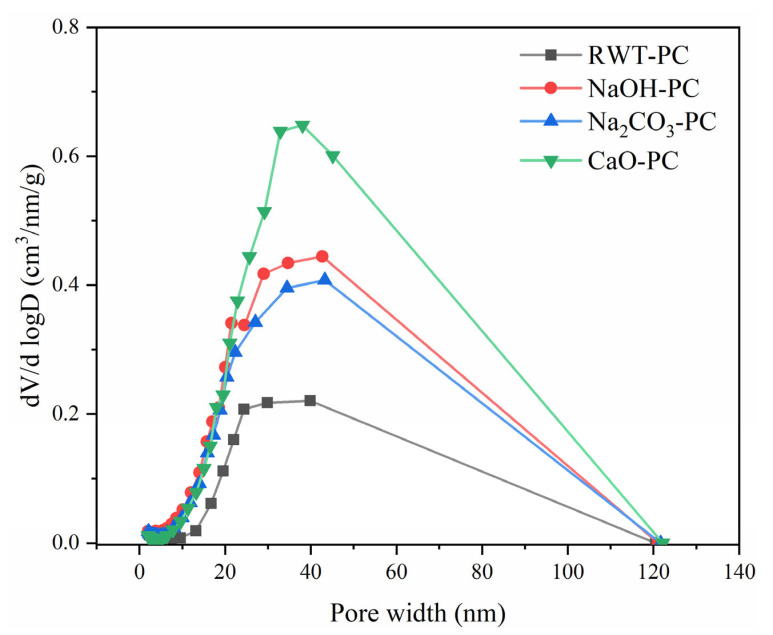
Pore distribution characterization of pyrolytic char (PC) derived from waste tires.

The N_2_ adsorption and desorption isotherms of PC derived from different catalytic pyrolysis processes are displayed in Figure 4. All the N_2_ adsorption and desorption curves resembled a Type IV isotherm with a hysteresis loop of the H3 type [49]. The Type IV isotherms showed a rapid rise in the medium relative pressure zone (0.3 < P/P_0_ < 0.8) and steep growth at high relative pressures (P/P_0_ > 0.9) owing to the capillary condensation phenomenon, thus representing mesopores as the main structure. Cesar et al. reported a similar finding in their research [50] where adsorbents from used tire rubber were used in the adsorption of organic and inorganic solutes in an aqueous solution. These results demonstrate that the adsorption effect of PC derived from waste tires could be attributed to porous adsorption. Furthermore, the pores of the PC were irregular, which could be a slit-type pore structure accumulated by particles [9]. Figure 5 shows the pore size distributions of RWT-PC, NaOH-PC, Na_2_CO_3_-PC, and CaO-PC. The distribution of micro- and mesopore diameters was evenly spread for all PCs according to the N_2_ desorption isotherms [49].

The multiple-point BET surface areas of RWT-PC, NaOH-PC, Na_2_CO_3_-PC, and CaO-PC were analyzed under isothermal liquid-nitrogen conditions [51]. The BET surface areas of NaOH-PC, Na_2_CO_3_-PC, and CaO-PC were improved; the relatively high surface area of NaOH-PC was 74.4 m^2^/g, whereas the surface areas of RWT-PC, Na_2_CO_3_-PC, and CaO-PC were 29.73, 64.95, and 67.31 m^2^/g, respectively (Table 3). The total pore volume exhibited the same variation trend as the surface area changed, but the change in the average pore diameter was the opposite. Therefore, it is evident that the addition of alkaline oxide had a positive effect on the pore structure of the PC derived from waste tires.

### 3.2. Effect of Parameters on Adsorption Performance

#### 3.2.1. Effect of Contact Time

**Figure 6:**
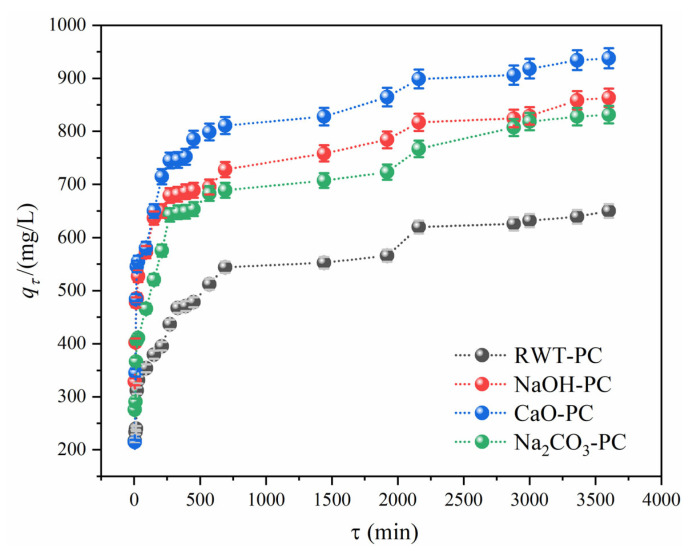
Effect of contact time on MG dye adsorption capacity (Dosage = 40 mg, MG concentration = 1000 mg/L, and T = 301 K).

Figure 6 shows the effect of contact time on the MG adsorption performance using different PCs. The uptake of MG by PCs showed that the sorption rate increased rapidly in the first 500 min and then slightly increased until saturation. NaOH-PC, CaO-PC, and Na_2_CO_3_-PC showed steeper curves, indicating faster adsorption than RWT-PC. The rapid adsorption in the first 500 min could be due to the availability of sufficient active sites on the surface, and the stronger affinity between MG and modified PCs promoting faster adsorption of NaOH-PC, CaO-PC, and Na_2_CO_3_-PC [52]. Therefore, the addition of NaOH, CaO, and Na_2_CO_3_ was beneficial for the enhancement of MG removal. The time to reach 500 mg/L was 600 min, which was faster than that of RWT-PC. Among these, CaO-PC had the highest adsorption performance for the MG solution, with an adsorption capacity of 937.8 mg/L, which was 1.5 times the equilibrium adsorption capacity of RWT-PC. This could be related to the larger pore volume and pore diameter of CaO-PC, providing more space for the adsorption and diffusion processes. Therefore, pore filling may be one of the main mechanisms of the adsorption of MG by tire pyrolytic char.

#### 3.2.2. Effect of Initial MG Concentration

**Figure 7:**
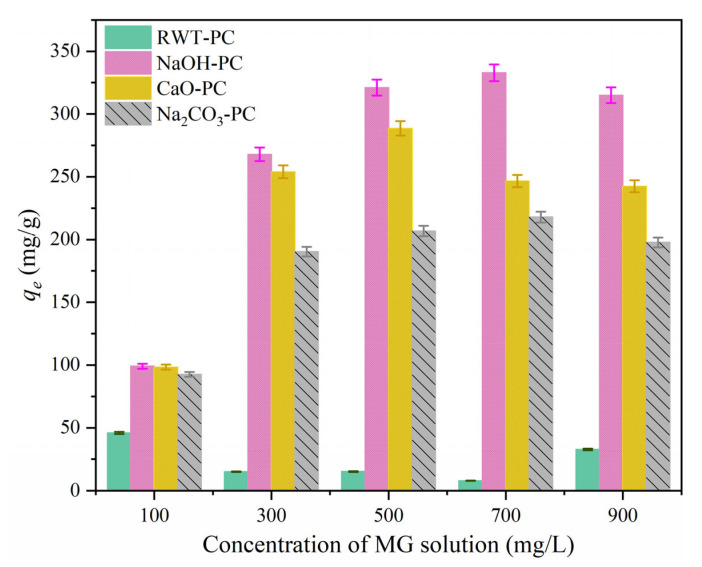
Effect of initial malachite green (MG) concentration on MG dye adsorption capacity (Dosage = 40 mg and T = 301 K).

Figure 7 shows the adsorption capacities of various PCs at initial MG concentrations of 100–900 mg/L. For the initial dye solution concentration of 100 mg/L, all four PCs showed a lower adsorption amount because the lower MG concentration offered fewer adsorbents, resulting in a large number of adsorption sites remaining on the PCs. With increased dye concentration, higher adsorption amounts were obtained, as shown in Figure 7. NaOH-PC and Na_2_CO_3_-PC showed a similar trend in the adsorption amount, reaching an adsorption capacity of 332.9 mg/g and 217.9 mg/g, respectively, when the initial MG concentration was 700 mg/L. RWT-PC and CaO-PC showed the highest adsorption capacities, 125.3 mg/g and 288.6 mg/g for the 500 mg/L MG, respectively. The adsorption capacity decreased when the initial concentration exceeded 700 mg/L. This was related to pore diffusion; a larger pore volume allowed enough space for MG molecules to diffuse from the solution to the pores of the PCs. However, the accumulated MG molecules blocked the pore structure of the PC. When the concentration continued to increase, the adsorption effect of the pore structure was weak, causing the adsorption amount to decrease [53]. For the same concentration solution, modified PCs showed a higher adsorption capacity than RWT-PC. The adsorption ability followed the order: NaOH-PC > CaO-PC > Na_2_CO_3_-PC > RWT-PC. Interestingly, this order was consistent with that of the BET surface area in the N_2_ adsorption-desorption test. This phenomenon could be attributed to the larger BET surface area available for more adsorption sites, therefore suggesting that a high MG concentration could be the driving force for the concentration gradient [54]. This would improve the adsorption to some extent. At a very high concentration, the reaction did not work.

#### 3.2.3. Effect of pH

**Figure 8:**
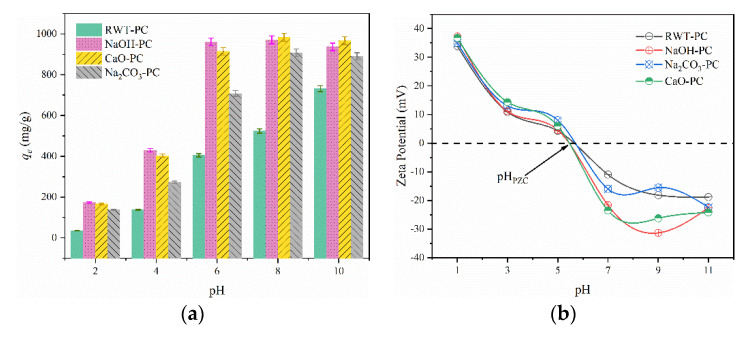
(**a**) Effect of pH on MG dye adsorption capacity (Dosage = 40 mg, MG concentration = 1000 mg/L, and T = 301 K) and (**b**) Zeta potential of four different adsorbents.

Solution pH is an important factor for adsorption [25]. Various dye pH solutions, ranging from 2–10, were chosen to measure the effect of pH on the adsorption amount of the PCs. All four PC adsorbents exhibited lower adsorption amounts in the acid solution. As the pH increased, more MG molecules were adsorbed onto the PCs, and the adsorption amount increased rapidly when the pH of the solution was greater than 4. When the pH was increased from 2 to 8, the adsorption amount of CaO-PC, NaOH-PC, and Na_2_CO_3_-PC increased from 166.4 mg/g, 173.3 mg/g, and 138.8 mg/g to 983.4 mg/g, 971.3 mg/g, and 907.8 mg/g, respectively. In an alkaline environment with a neutral pH, the four adsorbents exhibited similar adsorption capacities of the MG solution. This phenomenon can be explained by the zeta potentials at various pH values. As shown in Figure 8b, the zero-charge potentials (pH_PZC_) of the four adsorbents were in the range of 5–6. When the pH of the solution was lower than 6, the surfaces of the four adsorbents were positively charged, and MG, as a cationic dye, was easily decomposed under acidic conditions (p*Ka =* 10.3) [9]. The electrostatic repulsion of two positively charged materials in an aqueous solution results in competitive adsorption and a reduction in adsorption. At pH > 6, the negative surface of the adsorbents showed electrostatic attraction to the MG molecules; therefore, the adsorption amount increased rapidly. As the pH continued to increase, the adsorption capacity changed slightly, implying that the effect of the electrostatic force on the adsorption capacity was limited.

### 3.3. Adsorption Kinetics

The kinetics of the adsorption of MG onto the surface of RWT-PC, NaOH-PC, CaO-PC, and Na_2_CO_3_-PC were examined by conducting an equilibrium time investigation. The possible adsorption mechanism, pathway, and residence time to reach equilibrium can be predicted using kinetic models. Pseudo-first-order (PFO) and pseudo-second-order (PSO) models were used to evaluate the experimental kinetic data. The PFO model assumes that physical adsorption is the main method for limiting the adsorption rate, while the adsorption process based on chemisorption is more consistent with the PSO model.

The PFO equations given by Langergren and Svenska [55] were described as:

(2) $$ln\left(q_{e}-q_{\tau }\right)=\ln q_{e}-k_{1}\tau$$

(3) $$q_{\tau }=q_{e}\left(1-e^{-k_{1}\tau }\right)$$

where *q_e_* and *q_t_* are the equilibrium adsorption capacity (mg/g) and adsorption capacity at time t (mg/g) per unit mass of the adsorbent, respectively, and *k_1_* represents the adsorption rate constant (min^−1^). The values of the rate constant of Lagergren 1st order *k_1_* and *q_e_* were derived from the slopes and intercepts of the fitted nonlinear equation.

The nonlinear PSO expression based on equilibrium adsorption was as follows [56]:

(4) $$q_{\tau }=\frac{q_{e}^{2}k_{2}\tau }{1+q_{e}k_{2}\tau }$$

where *k*^2^ is the adsorption rate constant of PSO (g/mg·min). The derived values of the PSO model parameters, *k*^2^ and *q_e_*, were obtained from time-independent experimental data.

To better evaluate the fitting effect of the kinetic models, error functions were used to optimize the process as follows:

(5) $$R^{2}=1-\frac{\sum_{i-1}^{n}{\left(q_{e,exp}-q_{e,cal}\right)}^{2}}{\sum_{i=1}^{n}{\left(q_{e,exp}-\bar{q_{e,cal}}\right)}^{2}}$$

(6) $$RMSE=\sqrt{\frac{1}{n-2}\times \overset{n}{\underset{i=1}{\sum }}{\left(q_{e,exp}-q_{e,cal}\right)}^{2}}$$

**Figure 9:**
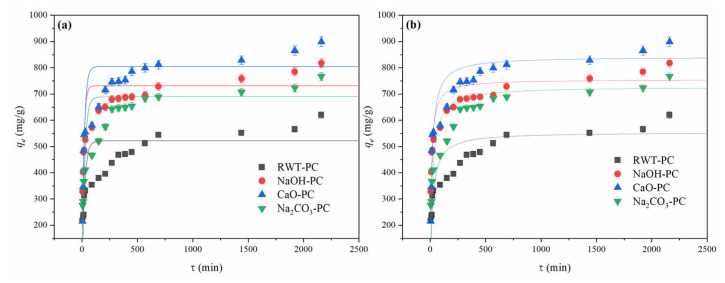
(**a**) Pseudo-first-order (PFO) and pseudo-second-order (PSO) (**b**) fitting curves of PCs derived from waste tires (Dosage = 40 mg, MG concentration = 1000 mg/L, and T = 301 K).

For the same initial MG concentration, the adsorption amounts *q_t_* on RWT-PC, NaOH-PC, CaO-PC, and Na_2_CO_3_-PC were recorded, and the fitting results for PFO and PSO are shown in Figure 9 and Table 4. The correlation coefficient *R*^2^ is an important indicator for measuring the matching degree of the PFO model. As shown in Table 4, the *R*^2^ value of PSO for all PCs was greater than that of PFO, indicating that the PSO model was more suitable for describing the adsorption process of MG onto PCs. Among them, CaO-PC had the highest *R*^2^, 0.914, indicating that chemisorption was the main adsorption method of CaO-PC. By adding small organics to test the adsorption characteristics of AC produced from the pyrolytic tire char, the process also followed the PSO model [23]. Meanwhile, the *q_e_* comparison between the calculated and experimental data showed that the PSO fitting results of *q_e, cal_* were closer to the actual equilibrium adsorption data. Root mean square error (*RMSE)* analysis was used to measure the deviation between the predicted value and the actual value, being sensitive to outliers in the data. The lower *RMSE* values of the PSO further demonstrated the adsorption mechanism of all PCs, where chemisorption was the primary adsorption kinetics. Among the four PC-derived adsorbents, NaOH-PC showed the lowest *RMSE* value for both the PFO and PSO models, indicating that, for the adsorbent, the fitted value of its adsorption data was closest to the true value. Therefore, the main mechanism of MG adsorption by the PCs was chemical adsorption.

An intraparticle diffusion (ID) model was applied to investigate the diffusion process. The equation of the ID model was as described below:

(7) $$q_{\tau }=k_{i}\tau^{\frac{1}{2}}+c$$

where *k_i_* is the intraparticle diffusion rate constant (mg/(g·min^1/2^)) and *c* is related to the boundary layer thickness on the adsorption rate.

**Figure 10:**
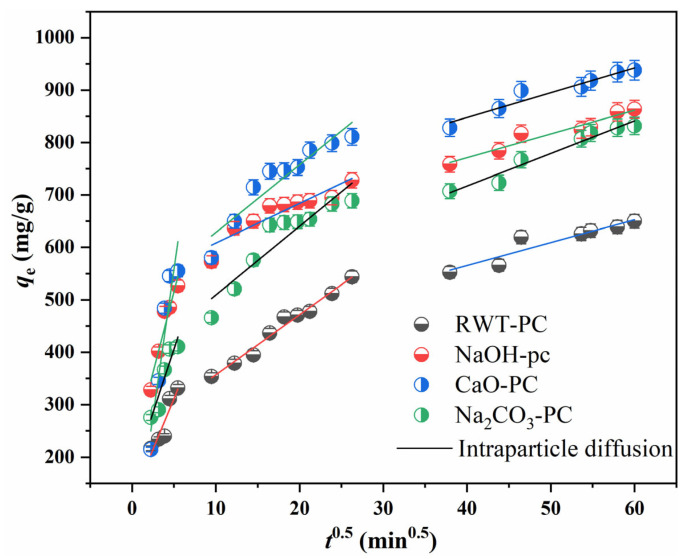
Intraparticle diffusion model of MG adsorption on PCs (Dosage = 40 mg, MG concentration = 1000 mg/L, and T = 301 K).

Figure 10 shows the linear fitting curves of the ID model, with all of the fitting lines divided into three parts: (1) MG molecules diffusing rapidly from the solution to the surface of the PCs, which could be related to the membrane diffusion; (2) the dye molecules entering the pore interior of the PCs and more MG cations accelerating in the pores, which was attributed to intraparticle diffusion; and (3) the pores and adsorption sites being occupied, resulting in a slow increase in adsorption capacity. All fitted curves had different intercept values (not passing through the origin), implying that both membrane diffusion and intraparticle diffusion occurred during the adsorption process.

### 3.4. Adsorption Isotherms

Isothermal adsorption evaluation is important for describing the interaction between an adsorbent and adsorbate [8]. There are several isothermal models to measure the adsorption process based on various assumptions. In this study, three different isotherms were applied to assess the adsorption data. The Langmuir, Freundlich, and Temkin isotherm models were fitted by nonlinear regression using Origin 2018 software. The fitted curves and data are presented in Figure 9 and Table 4, respectively.

The Langmuir model was used to investigate the relationship between *q_e_* and *C*_e_ at a temperature of 301 K using the equations displayed below (Equations (8) and (9)):

(8) $$q_{e}=\frac{q_{m}K_{L}C_{e}}{1+K_{L}C_{e}}$$

(9) $$\frac{C_{e}}{q_{e}}=\frac{1}{q_{m}K_{L}}+\frac{C_{e}}{q_{m}}$$

where *q_m_* (mg/g) represents the theoretical monolayer saturated adsorption capacity, *C_e_* (mg/L) is the adsorption equilibrium concentration, and *K_L_* is the Langmuir constant, which is related to the adsorption capacity of AC. A higher *K_L_* implies a stronger affinity between MG molecules and PCs.

The Freundlich equation is an empirical model that was applied linearly [9] in the invertible multilayer adsorption. Its linear and nonlinear expressions are shown below:

(10) $$q_{e}=K_{F}C_{e}^{\frac{1}{n}}$$

(11) $$lnq_{e}=\ln K_{F}+\frac{1}{n}lnC_{e}$$

where *K_F_* (mg/g·(L/mg)^1/n^) is the Freundlich equilibrium adsorption rate, and the value of *n* indicates the interaction strength between the adsorbent and adsorbate.

The Temkin model assumes that the decrease in adsorption heat is straight rather than logarithmic [57]. The expression for the model is given by Equations (12) and (13):

(12) $$q_{e}=\mathrm{RT}/\mathrm{bln}\left(K_{t}C_{e}\right)$$

(13) B=RT/b

where *R* is the gas constant, *T* is the Kelvin temperature scale (K), *K_t_* is the binding energy, *C_e_* is the equilibrium adsorption capacity of the adsorbate (mg/g), *b* is a constant related to the sorption ability, and B is the sorption heat correlation coefficient (J/mol).

**Figure 11:**
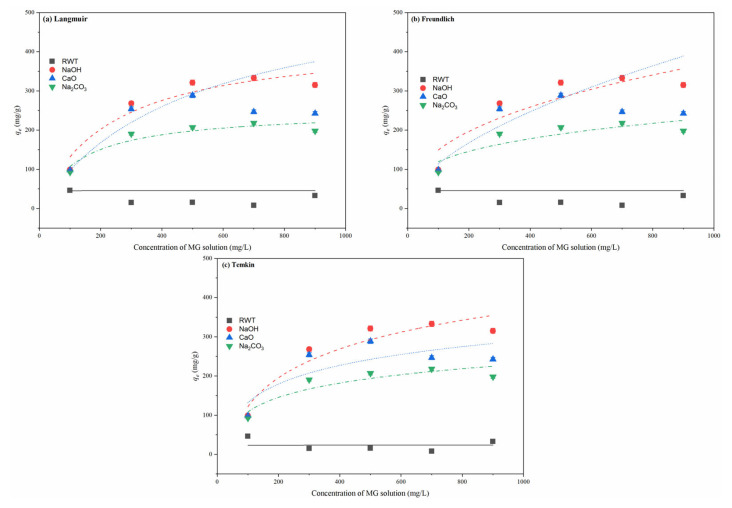
Adsorption isotherm fitting curves for MG sorption on PCs derived from waste tires (Dosage = 40 mg and T = 301 K).

Isothermal adsorption processes of RWT-PC, NaOH-PC, CaO-PC, and Na_2_CO_3_-PC were conducted, with Figure 11 showing the fitting results of the three models. The fitting curves followed the trend of the adsorption amount with the initial MG concentration, whereas for RWT-PC the adsorption amount increment was too low to fit the three isotherms well. The correlation coefficient *R*^2^ of all the isotherms for the four adsorbents followed the order: Langmuir > Freundlich > Temkin. This indicated that the Temkin model was the least suitable model to describe the adsorption of MG by PC, and the PCs’ adsorption methods onto MG were mainly monolayer adsorption. NaOH-PC exhibited the best compatibility with the Langmuir model of *R*^2^ = 0.918 (as shown in Table 5).

### 3.5. Comparison with Other Adsorbents

It is necessary to compare the prepared adsorbents with other adsorbents for MG adsorption [58]. In Table 6, some adsorbents derived from different biomass materials and treated with various activators are listed for comparison with the adsorption results of this study. It can be observed that the RWT-PC had a higher equilibrium adsorption capacity than other adsorbents. The highest adsorption capacity was 937.8 mg/g in CaO-PC. Most of the listed adsorbents followed the PSO model fitting and the Langmuir model. These results demonstrate that the adsorption pathway is a monolayer adsorption based on chemical adsorption.

## 4. Conclusions

Pyrolytic chars (PC) derived from waste tires activated with NaOH, Na_2_CO_3_, and CaO were investigated for removing MG from aqueous solutions. PCs exhibit excellent structural properties and surface chemistry. A large number of functional groups dispersed on the surface of PC is beneficial for MG adsorption, and the high BET surface area offers more adsorption sites. The maximum adsorption capacity was 937.8 mg/g at 301 K, a pH 6.8, and an initial MG concentration of 1000 mg/L. The kinetics study showed that the pseudo-second-order model best fit the experimental data. An isothermal investigation of PC adsorption on the MG solution demonstrated the best match with the Langmuir isotherm. This work offers an efficient method for the disposal of waste rubber and the removal of organic dye solutions.

## Figures and Tables

**Table 1:** Elemental content analysis of waste tires (daf.).

Ultimate Analysis				*S*_t,d_
C	H	O ^a^	N	
63.859	5.305	5.019	1.181	1.299

^a^ By difference.

**Table 2:** The relative content of the surface functional groups is determined by C1s of various pyrolytic chars.

Samples	C-C		C-O		O-C=O	
	B.E. (eV)	Concentration	B.E. (eV)	Concentration	B.E. (eV)	Concentration
RWT-PC	284.43	73.53%	285.07	22.06%	289.32	4.41%
NaOH-PC	284.43	65.79%	285.43	24.34%	289.77	9.87%
Na_2_CO_3_-PC	284.38	55.56%	285.01	36.11%	288.11	8.33%
CaO-PC	284.48	68.49%	285.39	22.60%	289.82	8.90%

**Table 3:** Structural parameters of pyrolytic char (PC) derived from waste tires.

Sample	BET Surface (m^2^/g)	Total Pore Volume (cm^3^/g)	Average Pore Diameter (nm)
RWT-PC	29.73	0.24	29.43
NaOH-PC	74.44	0.52	27.71
Na_2_CO_3_-PC	64.95	0.47	28.96
CaO-PC	67.31	0.62	36.95

**Table 4:** Kinetic parameters of MG adsorption on PCs derived from waste tire.

Kinetic Models	Parameters	Pyrolytic Chars			
		RWT-PC	NaOH-PC	CaO-PC	Na_2_CO_3_-PC
	*q_e,exp_* (mg/g)	631.7	858.5	937.8	831.7
PFO	*q_e,cal_* (mg/g)	480.0	692.0	763.5	642.0
	*k*_1_(min^−1^)	0.0533	0.0765	0.0583	0.0518
	*R*^2^ (nonlinear)	0.645	0.754	0.837	0.736
	*RMSE*	22.045	20.1	22.705	24.332
PSO	*q_e,cal_* (mg/g)	508.7	717.6	802.6	677.6
	*k*^2^×10^4^ (g/mg·min)	1.322	1.667	0.965	1.008
	*R*^2^ (nonlinear)	0.779	0.884	0.914	0.866
	*RMSE*	20.303	15.352	18.934	20.197
ID	*K_i,1_* (mg/g min^0.5^)	118.2	207.3	111.5	164.3
	*RMSE*	34.916	37.680	22.499	41.084
	*C_1_*	38.65	61.61	0.11	48.31
	*R*^2^ (linear)	0.867	0.934	0.891	0.881
	*K_i,_*^2^ (mg/g min^0.5^)	242.4	532.2	499.1	377.9
	*RMSE*	11.141	21.600	31.951	36.909
	*C* ^2^	11.46	7.59	12.92	13.12
	*R*^2^ (linear)	0.981	0.861	0.891	0.864
	*K_i,3_* (mg/g min^0.5^)	390.5	589.9	658.4	468.3
	*RMSE*	36.203	25.743	27.843	28.915
	*C_3_*	4.37	4.53	4.73	6.22
	*R*^2^ (linear)	0.884	0.942	0.938	0.960

**Table 5:** Isothermal adsorption fitting parameters of MG on PCs.

Isotherms	Parameters	PCs			
		RWT-PC	NaOH-PC	CaO-PC	Na_2_CO_3_-PC
Langmuir	*q_L_* (mg/g)	45.633	432.573	579.272	251.013
	*K_L_* (L/mg)	0.65450	0.00438	0.00204	0.00748
	*R* ^2^	/	0.918	0.758	0.896
Freundlich	K_F_ (mg/g(L/mg)^1/n^ )	45.386	24.061	8.633	31.748
	n	2.26	2.52	1.79	3.47
	*R* ^2^	/	0.745	0.703	0.671
Temkin	b	/	0.03161	0.06817	0.07789
	K_T_(L/mg)	/	105.968	68.697	52.896
	*R* ^2^	/	0.899	0.544	0.770

**Table 6:** Comparison of MG adsorption capacity on different biomass-based adsorbents.

Raw Material	Activator	Adsorption Capacity (mg/g)	Kinetics	Isotherms	Reference
*Luffa aegyptica* peel	NaOH	78.79	PSO	Langmuir	[16]
Rice husk	NaOH	373.02	PSO	/	[17]
*Pinus roxburghii* cone	Acetic acid	250	/	Langmuir	[18]
Chinese Fan Palm Seed	/	21.4	/	Langmuir	[43]
Nutraceutical industrial fenugreek seed spent	/	130	PSO	Langmuir	[44]
Rubber seed shells	H_3_PO_4_	58.48	PSO	Freundlich	[45]
Rubber seed shells	NaCl	56.82	PSO	Freundlich	[45]
Waste tires	/	631.7	PSO	Langmuir	This study
Waste tires	NaOH	858.5	PSO	Langmuir	This study
Waste tires	CaO	937.8	PSO	Langmuir	This study
Waste tires	Na_2_CO_3_	831.7	PSO	Langmuir	This study

## Data Availability

All data are contained within the article.
